# Supplementary material for: Nimodipine Treatment Protects Auditory Hair Cells from Cisplatin-Induced Cell Death Accompanied by Upregulation of LMO4
Source: Int J Mol Sci. 2022 May 21;23(10):5780. doi: 10.3390/ijms23105780 (PMC9145067; doi:10.3390/ijms23105780)
Supplement: Supplementary file 1 [file ijms-23-05780-s001.zip › Table S1.pdf]

Table S1. Effect of nimodipine pre-treatment on cell death of hair cells during stress induction by cisplatin

Treatment		UB/OC-1, undiff. Mean Diff. (95 % CI) *	UB/OC-2, undiff. Mean Diff. (95 % CI) *	UB/OC-1, diff. Mean Diff. (95 % CI) *	UB/OC-2, diff. Mean Diff. (95 % CI) *
MEM + INF-γ/0.9 % NaCl	MEM + INF-γ/20 µM C	-13.1 (-28.0 to 1.9)	-25.7 (-33.8 to -17.7) *	-46.4 (-77.4 to -15.3) *	-42.1 (-82.1 to -2.1) *
	MEM + INF-γ/50 µM C	-19.3 (-34.2 to -4.4) *	-22.1 (-30.2 to -14.1) *	-53.2 (-85.0 to -22.9) *	-24.0 (-64.0 to 16.0)
	MEM + INF-γ/100 µM C	-22.1 (-37.0 to -7.2)	-31.1 (-39.1 to -23.1) *	-83.8 (-114.8 to 52.7) *	-79.1 (-119.1 to -39.1) *
	EtOH/0.9 % NaCl	0.8 (-14.1 to 15.8)	0.8 (-7.2 to 8.9)	2.6 (-28.4 to 33.7)	0.5 (-39.5 to 40.5)
	EtOH/20 µM C	-11.7 (-26.6 to 3.2)	-24.3 (-32.3 to -16.3) *	-39.6 (-70.7 to -8.6) *	-42.2 (-82.3 to -2.2) *
	EtOH/50 µM C	-16.7 (-31.6 to -1.7) *	-20.5 (-28.5 to -12.4) *	-49.7 (-80.7 to -18.6) *	-19.7 (-59.7 to 20.3)
	EtOH/100 µM C	-21.2 (-36.2 to -6.3) *	-25.7 (-33.7 to -17.7) *	-77.7 (-108.7 to -46.6) *	-71.3 (-111.3 to -31.3) *
	10 µM N/0.9% NaCl	2.1 (-12.8 to 17.1)	1.7 (-6.3 to 9.8)	-4.8 (-35.8 to 26.3)	-28.3 (-68.3 to 11.7)
	10 µM N/20 µM C	-6.3 (-21.2 to 8.6)	-14.1 (-22.2 to -6.1) *	-23.8 (-54.8 to 7.3)	-28.5 (-68.5 to 11.5)
	10 µM N/50 µM C	-8.4 (-23.3 to 6.5)	-10.9 (-18.9 to -2.9) *	-36.1 (-67.1 to -5.0) *	-16.3 (-56.3 to 23.7)
	10 µM Nimo/100 µM C	-7.2 (-22.1 to 7.8)	-14.3 (-22.4 to -6.3) *	-51.8 (-82.9 to -20.7) *	-52.9 (-92.9 to -12.9) *
	20 µM Nimo/0.9 % NaCl	0.5 (-14.4 to 15.4)	0.9 (-7.2 to 8.9)	-3.2 (-34.3 to 27.8)	-30.0 (-70.0 to 10.0)
	20 µM N/20 µM C	-3.4 (-18.3 to 11.5)	-9.3 (-17.4 to -1.3) *	-17.4 (-48.5 to 13.6)	-26.2 (-66.3 to 13.8)
	20 µM N/50 µM C	-6.8 (-21.7 to 8.2)	-9.9 (-17.9 to -1.9) *	-26.8 (-57.9 to 4.2)	-15.5 (-55.5 to 24.5)
	20 µM N/100 µM C	-4.7 (-19.6 to 10.3)	-9.2 (-17.3 to -1.2) *	-41.1 (-72.1 to 10.0) *	-49.1 (-89.1 to -9.0) *
MEM + INF-γ/20 µM	MEM + INF-γ/50 µM C	-6.2 (-21.2 to 8.7)	3.6 (-4.5 to 11.6)	-7.6 (-38.6 to 23.5)	18.2 (-21.8 to 58.2)
	MEM + INF-γ/100 µM C	-9.1 (-24.0 to 5.9)	-5.4 (-13.4 to 2.7)	-37.4 (-68.5 to -6.3) *	-37.0 (-77.0 to 3.0)
	EtOH/0.9 % NaCl	13.9 (-1.1 to 28.8)	26.5 (18.5 to 34.6) *	49.0 (17.9 to 80.0) *	42.6 (2.6 to 82.6) *
	EtOH/20 µM C	1.4 (-13.6 to 16.3)	1.4 (-6.6 to 9.5)	6.7 (-24.3 to 37.8)	-0.1 (-40.1 to 39.9)
	EtOH/50 µM C	-3.6 (-18.6 to 11.3)	5.3 (-2.8 to 13.3)	-3.3 (-34.3 to 27.8)	22.4 (-17.6 to 62.5)
	EtOH/100 µM C	-8.2 (-23.1 to 6.8)	0.0 (-8.0 to 8.1)	-31.3 (-62.4 to -0.2) *	-29.1 (-69.1 to 10.9)
	10 µM N/0.9% NaCl	15.2 (0.2 to 30.1) *	27.5 (19.4 to 35.5) *	41.6 (10.5 to 72.6) *	13.9 (-26.1 to 53.9)
	10 µM N/20 µM C	6.8 (-8.2 to 21.7)	11.6 (3.6 to 19.6) *	22.6 (-8.5 to 53.7)	13.6 (-26.4 to 53.7)
	10 µM N/50 µM C	4.6 (-10.3 to 19.6)	14.8 (6.8 to 22.8) *	10.3 (-20.8 to 41.4)	25.8 (-14.2 to 64.8)
	10 µM N/100 µM C	5.9 (-9.0 to 20.8)	11.4 (3.4 to 19.4) *	-5.4 (-36.5 to 25.6)	-10.8 (-50.8 to 29.2)
	20 µM N/0.9 % NaCl	13.6 (-1.4 to 28.5)	26.6 (18.5 to 34.6) *	43.1 (12.1 to 74.2) *	12.1 (-27.9 to 52.1)
	20 µM N/20 µM C	9.6 (-5.3 to 24.6)	16.4 (8.4 to 24.4) *	29.0 (-2.1 to 60.0)	15.9 (-24.1 to 55.9)
	20 µM N/50 µM C	6.3 (-8.7 to 21.2)	15.8 (7.8 to 23.8) *	19.5 (-11.5 to 50.6)	26.6 (-13.4 to 66.6)
	20 µM N/100 µM C	8.4 (-6.6 to 23.3)	16.5 (8.5 to 24.5) *	5.3 (-25.8 to 36.4)	-6.9 (-46.9 to 33.1)
MEM + INF-γ/50 µM C	MEM + INF-γ/100 µM C	-2.8 (-17.7 to 12.1)	-9.0 (-17.0 to -0.9) *	-29.8 (-60.9 to 1.2)	-55.2 (-95.2 to -15.2) *
	EtOH/0.9 % NaCl	20.1 (5.2 to 35.1) *	23.0 (14.9 to 31.0) *	56.5 (25.5 to 87.6) *	24.5 (-15.6 to 64.5)
	EtOH/20 µM C	7.6 (-7.3 to 22.5)	-2.2 (-10.2 to 5.9)	14.3 (-16.8 to 45.3)	-18.3 (-58.3 to 21.7)
	EtOH/50 µM C	2.6 (-12.3 to 17.6)	1.7 (-6.3 to 9.7)	4.3 (-26.8 to 35.3)	4.3 (-35.7 to 44.3)
	EtOH/100 µM C	-1.9 (-16.9 to 13.0)	-3.6 (-11.6 to 4.5)	-23.7 (-54.8 to 7.3)	-47.3 (-87.3 to -7.3) *
	10 µM N/0,9% NaCl	21.4 (6.5 to 36.4) *	23.9 (15.8 to 31.9) *	49.1 (18.1 to 80.2) *	-4.3 (-44.3 to 35.7)
	10 µM N/20 µM C	13.0 (-1.9 to 27.9)	8.0 (0.0 to 16.1)	30.2 (-0.9 to 61.2)	-4.5 (-44.5 to 35.5)
	10 µM N/50 µM C	10.9 (-4.1 to 25.8)	11.2 (3.2 to 19.3) *	17.9 (-13.2 to 48.9)	7.6 (-32.4 to 47.6)
	10 µM N/100 µM C	12.1 (-2.8 to 27.1)	7.8 (-0.2 to 15.9)	2.1 (-28.9 to 33.2)	-29.0 (-69.0 to 11.0)
	20 µM N/0.9 % NaCl	19.8 (4.9 to 34.7) *	23.0 (15.0 to 31.1) *	50.7 (19.6 to 81.7) *	-6.0 (-46.0 to 34.0)
	20 µM N/20 µM C	15.9 (0.9 to 30.8) *	12.8 (4.8 to 20.9) *	36.5 (5.5 to 67.6) *	-2.3 (-42.3 to 37.7)
	20 µM N/50 µM C	12.5 (-2.4 to 27.4)	12.2 (4.2 to 20.3) *	27.1 (-4.0 to 58.2)	8.4 (-31.6 to 48.4)
	20 µM N/100 µM C	14.6 (-0.3 to 29.6)	12.9 (4.9 to 21.0) *	12.9 (-18.2 to 43.9)	-25.1 (-65.1 to 14.9)
MEM + INF-γ/100 µM C	EtOH/0.9 % NaCl	22.9 (8.0 to 37.9) *	31.9 (23.9 to 40.0) *	86.4 (55.3 to 117.4) *	79.6 (39.6 to 119.6) *
	EtOH/20 µM C	10.4 (-4.5 to 25.4)	6.8 (-1.2 to 14.8)	44.1 (13.1 to 75.2) *	36.9 (-3.1 to 76.9)
	EtOH/50 µM C	5.4 (-9.5 to 20.4)	10.6 (2.6 to 18.7) *	34.1 (3.0 to 65.2) *	59.4 (19.4 to 99.4) *
	EtOH/100 µM C	0.9 (-14.1 to 15.8)	5.4 (-2.6 to 13.4)	6.1 (-25.0 to 37.1)	7.9 (-32.1 to 47.9)
	10 µM N/0,9% NaCl	24.2 (9.3 to 39.2) *	32.8 (24.8 to 40.9) *	79.0 (47.9 to 110.0) *	50.8 (10.8 to 90.8) *
	10 µM N/20 µM C	15.8 (0.9 to 30.8) *	17.0 (8.9 to 25.0) *	60.0 (28.9 to 91.1) *	50.6 (10.6 to 90.6) *
	10 µM N/50 µM C	13.7 (-1.2 to 28.6)	20.2 (12.2 to 28.2) *	47.7 (16.6 to 78.8) *	62.8 (22.8 to 102.8) *
	10 µM N/100 µM C	15.0 (0.0 to 29.9) *	16.8 (8.7 to 24.8) *	32.0 (0.9 to 63.0) *	26.2 (-13.8 to 66.2)
	20 µM N/0.9 % NaCl	22.6 (7.7 to 37.6) *	32.0 (23.9 to 40.0) *	80.5 (49.5 to 111.6) *	49.1 (9.1 to 89.2) *
	20 µM N/20 µM C	18.7 (3.8 to 33.6) *	21.8 (13.7 to 29.8) *	66.3 (35.3 to 97.4) *	52.9 (12.9 to 92.9) *
	20 µM N/50 µM C	15.3 (0.4 to 30.3) *	21.2 (13.1 to 29.2) *	56.9 (25.9 to 88.0) *	63.6 (23.6 to 103.6) *
	20 µM N/100 µM C	17.4 (2.5 to 32.4) *	21.9 (13.8 to 29.9) *	42.7 (11.6 to 73.8) *	30.1 (-10.0 to 70.1)
EtOH/0.9 % NaCl	EtOH/20 µM C	-12.5 (-27.5 to 2.4)	-25.1 (-33.2 to -17.1) *	-42.3 (-73.3 to -11.2) *	-42.7 (-82.7 to -2,7) *
	EtOH/50 µM C	-17.5 (-32.4 to -2.6)	-21.3 (-29.1 to -13.2) *	-52.3 (-83.3 to -21.2) *	-20.2 (-60.2 to 19.8)
	EtOH/100 µM C	-22.1 (-37.0 to -7.1)	-26.5 (-34.6 to -18.5) *	-80.3 (-111.4 to -49.2) *	-71.7 (-111.8 to -31.7) *
	10 µM N/0.9% NaCl	1.3 (-13.6 to 16.2)	0.9 (-7.1 to 9.0)	-7.4 (-38.4 to 23.7)	-28.8 (-68.8 to 11.2)
	10 µM N/20 µM C	-7.1 (-22.1 to 7.8)	-15.0 (-23.0 to -6.9) *	-26.4 (-57.4 to 4.7)	-29.0 (-69.0 to 11.0)
	10 µM N/50 µM C	-9.2 (-24.2 to 5.7)	-11.7 (-19.8 to -3.7) *	-38.7 (-69.7 to -7.6) *	-16.8 (-56.8 to 23.2)
	10 µM N/100 µM C	-8.0 (-22.9 to 7.0)	-15.1 (-23.2 to -7.1) *	-54.5 (-85.5 to -23.4) *	-53.4 (-93.4 to -13.4) *
	20 µM N/0.9 % NaCl	-0.3 (-15.2 to 14.6)	0.0 (-8.0 to 8.1)	-5.9 (-36.9 to 25.2)	-30.5 (-70.5 to 9.5)
	20 µM N/20 µM C	-4.2 (-19.2 to 19.7)	-10.1 (-18.2 to -2.1) *	-20.0 (-51.1 to 11.0)	-26.7 (-66.7 to 13.3)
	20 µM N/50 µM C	-7.6 (-22.5 to 7.3)	-10.7 (-18.8 to -2.7) *	-29.4 (-60.5 to 1.6)	-16.0 (-56.0 to 24.0)
	20 µM N/100 µM C	-5.5 (-20.4 to 9.4)	-10.0 (-18.1 to -2.0) *	-43.7 (-74.7 to -12.6) *	-49.5 (-89.6 to -9.5) *
EtOH/20 µM C	EtOH/50 µM C	-5.0 (-19.9 to 9.9)	3.9 (-4.2 to 11.9)	-10.0 (-41.1 to 21.0)	22.6 (-17.5 to 62.6)

	EtOH/100 µM C	-9.5 (-24.5 to 5.4)	-1.4 (-9.4 to 6.6)	-38.0 (-69.1 to -7.0) *	-29.0 (-69.0 to 11.0)
	10 µM N/0,9% NaCl	13.8 (-1.1 to 28.7)	26.0 (18.0 to 34.1) *	34.9 (3.8 to 65.9) *	14.0 (-26.0 to 54.0)
	10 µM N/20 µM C	5.4 (-9.5 to 20.3)	10.2 (2.1 to 18.2) *	15.9 (-15.2 to 46.9)	13.8 (-26.3 to 53.8)
	10 µM N/50 µM C	3.3 (-11.7 to 18.2)	13.4 (5.4 to 21.4) *	3.6 (-27.5 to 34.6)	25.9 (-14.1 to 65.1)
	10 µM N/100 µM C	4.5 (-10.4 to 19.5)	10.0 (2.0 to 18.0) *	-12.1 (-43.2 to 18.9)	-10.7 (-50.7 to 29.3)
	20 µM N/0.9 % NaCl	12.2 (-2.7 to 27.1)	25.2 (17.1 to 33.2) *	36.4 (5.3 to 67.5) *	12.2 (-27.8 to 52.3)
	20 µM N/20 µM C	8.3 (-6.7 to 23.2)	15.0 (6.9 to 23.0) *	22.2 (-8.8 to 53.3)	16.0 (-24.0 to 56.0)
	20 µM N/50 µM C	4.9 (-10.0 to 19.8)	14.4 (6.4 to 22.4) *	12.8 (-18.2 to 43.9)	26.7 (-13.3 to 66.7)
	20 µM N/100 µM C	7.0 (-7.9 to 21.9)	15.1 (7.1 to 23.1) *	-1.4 (-32.5 to 29.6)	-6.8 (-46.8 to 33.2)
EtOH/50 µM C	EtOH/100 µM C	-4.6 (-19.5 to 10.4)	-5.2 (-13.3 to 2.8)	-28.0 (-59.1 to 3.0)	-51.6 (-91.6 to -11.6) *
	10 µM N/0,9% NaCl	18.8 (3.9 to 33.7) *	22.2 (14.2 to 30.2) *	44.9 (13.8 to 75.9) *	-8.6 (-48.6 to 31.4)
	10 µM N/20 µM C	10.4 (-4.5 to 25.3)	6.3 (-1.7 to 14.4)	25.9 (-5.2 to 56.9)	-8.8 (-48.8 to 31.2)
	10 µM N/50 µM C	8.3 (-6.7 to 23.2)	9.5 (1.5 to 17.6) *	13.6 (-17.5 to 44.6)	3.4 (-36.6 to 43.4)
	10 µM N/100 µM C	9.5 (-5.4 to 24.5)	6.1 (-1.9 to 14.2)	-2.1 (-33.2 to 28.9)	-33.2 (-73.2 to 6.8)
	20 µM N/0.9 % NaCl	17.2 (2.3 to 32.1) *	21.3 (13.3 to 29.4) *	46.4 (15.3 to 77.5) *	-10.3 (-50.3 to 29.7)
	20 µM N/20 µM C	13.3 (-1.7 to 28.2)	11.1 (3.1 to 19.2) *	32.2 (1.2 to 63.3) *	-6.6 (-46.6 to 33.4)
	20 µM N/50 µM C	9.9 (-5.0 to 24.8)	10.5 (2.5 to 18.6) *	22.8 (-8.2 to 53.9)	4.2 (-35.8 to 44.2)
	20 µM N/100 µM C	12.0 (-2.9 to 26.9)	11.2 (3.2 to 19.3) *	8.6 (-22.5 to 39.6)	-29.4 (-69.4 to 10.6)
EtOH/100 µM C	10 µM N/0,9% NaCl	23.4 (8.4 to 38.3)	27.4 (19.4 to 35.5) *	72.9 (41.8 to 104.0) *	43.0 (3.0 to 83.0) *
	10 µM N/20 µM C	14.9 (0.0 to 29.9) *	11.6 (3.5 to 19.6) *	53.9 (22.8 to 85.0) *	42.8 (2.8 to 82.8) *
	10 µM N/50 µM C	12.8 (-2.1 to 27.8)	14.8 (6.8 to 22.8) *	41.6 (10.6 to 72.7) *	54.9 (14.9 to 94.9) *
	10 µM N/100 µM C	14.1 (-0.9 to 29.0)	11.4 (3.3 to 19.4) *	25.9 (-5.2 to 56.9)	18.3 (-21.7 to 58.3)
	20 µM N/0.9 % NaCl	21.7 (6.8 to 36.7) *	26.6 (18.5 to 34.6) *	74.4 (43.4 to 105.5) *	41.3 (1.2 to 81.3) *
	20 µM N/20 µM C	17.8 (2.9 to 32.7) *	16.4 (8.3 to 24.4) *	60.3 (29.2 to 91.3) *	45.0 (5.0 to 85.0) *
	20 µM N/50 µM C	14.4 (-0.5 to 29.4)	15.8 (7.8 to 23.8) *	50.8 (19.8 zo 81.9) *	55.7 (15.7 to 95.7) *
	20 µM N/100 µM C	16.6 (1.6 to 31.5) *	16.5 (8.4 to 24.5) *	36.6 (5.6 to 67.7) *	22.2 (-17.8 to 62.2)
10 µM Nimo/0,9% NaCl	10 µM N/20 µM C	-8.4 (-23.3 to 6.5)	-15.9 (-23.9 to -7.8) *	-19.0 (-50.0 to 12.1)	-0.2 (-40.2 to 39.8)
	10 µM N/50 µM C	-10.5 (-25.5 to 4.4)	-12.6 (-20.7 to -4.6) *	-31.3 (-62.3 to -0.2) *	11.9 (-28.1 to 52.0)
	10 µM N/100 µM C	-9.3 (-24.2 to 5.7)	-16.1 (-24.1 to -8.0) *	-47.0 (-78.1 to -16.0) *	-24.6 (-64.7 to 15.4)
	20 µM N/0.9 % NaCl	-1.6 (-16.5 to 13.3)	-0.9 (-8.9 to 7.2)	1.5 (-29.5 to 32.6)	-1.7 (-41.7 to 38.3)
	20 µM N/20 µM C	-5.5 (-20.5 to 9.4)	-11.1 (-19.1 to -3.0) *	-12.6 (-43.7 to 18.4)	2.0 (-38.0 to 42.0)
	20 µM N/50 µM C	-8.9 (-23.8 to 6.0)	-11.6 (-19.7 to -3.6) *	-22.0 (-53.1 to 9.0)	12.8 (-27.2 to 52.8)
	20 µM N/100 µM C	-6.8 (-21.7 to 8.1)	-11.0 (-19.0 to -2.9) *	-36.3 (-67.3 to -5.2) *	-20.8 (-60.8 to 19.2)
10 µM N/20 µM C	10 µM N/50 µM C	-2.1 (-17.1 to 12.8)	3.2 (-4.8 to 11.3)	-12.3 (-43.4 to 18.8)	12.2 (-27.8 to 52.2)
	10 µM N/100 µM C	-0.9 (-15.8 to 14.1)	-0.2 (-8.2 to 7.9)	-28.0 (-59.1 to 3.0)	-24.4 (-64.4 to 15.6)
	20 µM N/0.9 % NaCl	6.8 (-8.1 to 21.7)	15.0 (7.0 to 23.0) *	20.5 (-10.5 to 51.6)	-1.5 (-41.5 to 38.5)
	20 µM N/20 µM C	2.9 (-12.1 to 17.8)	4.8 (-3.2 to 12.8)	6.4 (-24.7 to 37.4)	2.3 (-37.8 to 42.3)
	20 µM N/50 µM C	-0.5 (-15.4 to 14.4)	4.2 (-3.8 to 12.3)	-3.1 (-34.1 to 28.0)	13.0 (-27.0 to 53.0)
	20 µM N/100 µM C	1.6 (-13.3 to 16.5)	4.9 (-3.1 to 13.0)	-17.3 (-48.4 to 13.8)	-20.6 (-60.6 to 19.4)
10 µM N/50 µM C	10 µM N/100 µM C	1.3 (-13.7 to 16.2)	-3.4 (-11.4 to 4.6)	-15.7 (-46.8 to 15.3)	-36.6 (-76.6 to 3.4)
	20 µM N/0.9 % NaCl	8.9 (-6.0 to 23.9)	11.8 (3.7 to 19.8) *	32.8 (1.8 to 63.9) *	-13.7 (-53.7 to 26.3)
	20 µM N/20 µM C	5.0 (-9.9 to 19.9)	1.6 (-6.5 to 9.6)	18.7 (-12.4 to 49.7)	-9.9 (-49.9 to 30.1)
	20 µM N/50 µM C	1.6 (-13.3 to 16.6)	1.0 (-7.0 to 9.0)	9.2 (-21.8 to 40.3)	0.8 (-39.2 to 40.8)
	20 µM N/100 µM C	3.7 (-11.2 to 18.7)	1.7 (-6.3 to 9.7)	-5.0 (-36.1 to 26.1)	-32.7 (-72.7 to 7.3)
10 µM N/100 µM C	20 µM N/0.9 % NaCl	7.7 (-7.3 to 22.6)	15.2 (7.1 to 23.2) *	48.5 (17.5 to 79.6) *	22.9 (-17.1 to 62.9)
	20 µM N/20 µM C	3.7 (-11.2 to 18.7)	5.0 (-3.0 to 13.0)	34.4 (3.3 to 65.4)	26.7 (-13.3 to 66.7)
	20 µM N/50 µM C	0.4 (-14.6 to 15.3)	4.4 (-3.6 to 12.4)	25.0 (-6.1 to 56.0)	37.4 (-2.6 to 77.4)
	20 µM N/100 µM C	2.5 (-12.5 to 17.4)	5.1 (-2.9 to 13.1)	10.7 (-20.3 to 41.8)	3.9 (-36.1 to 43.9)
20 µM N/0.9 % NaCl	20 µM N/20 µM C	-3.9 (-18.9 to 11.0)	-10.2 (-18.2 to -2.2) *	-14.1 (-45.1 to 16.9)	3.8 (-36.3 to 43.8)
	20 µM N/50 µM C	-7.3 (-22.2 to 7.6)	-10.9 (-18.8 to -2.7) *	-23.6 (-54.6 to 7.5)	14.5 (-25.5 to 54.5)
	20 µM N/100 µM C	-5.2 (-20.1 to 9.7)	-10.1 (-18.1 to -2.0) *	-37.8 (-68.9 to -6.8) *	-19.1 (-59.1 to 21.0)
20 µM N/20 µM C	20 µM N/50 µM C	-3.4 (-18.3 to 11.6)	-0.6 (-8.6 to 7.4)	-9.4 (-40.5 to 21.6)	10.7 (-29.3 to 50.7)
	20 µM N/100 µM C	-1.3 (-16.2 to 13.7)	0.1 (-7.9 to 8.1)	-23.7 (-54.7 to 7.4)	-22.8 (-62.8 to 17.2)
20 µM N/50 µM C	20 µM N/100 µM C	2.1 (-12.8 to 17.0)	0.7 (-7.3 to 8.7)	-14.2 (-45.3 to 16.8)	-33.5 (-73.5 to 6.5)

undiff., undifferentiated; diff., differentiated; Mean Diff., Mean Difference; CI., confidence interval; N., nimodipine; C., cisplatin
