# Supplementary material for: Nimodipine Treatment Protects Auditory Hair Cells from Cisplatin-Induced Cell Death Accompanied by Upregulation of LMO4
Source: Int J Mol Sci. 2022 May 21;23(10):5780. doi: 10.3390/ijms23105780 (PMC9145067; doi:10.3390/ijms23105780)
Supplement: Supplementary file 1 [file ijms-23-05780-s001.zip › Table S2.pdf]

**Table S2. Effect of nimodipine pre-treatment on intracellular calcium concentration in hair cells under stress induced by cisplatin**

| | | UB/OC-1, undiff. | UB/OC-2, undiff. |
|---|---|---|---|
| Treatment | | **Mean Diff.** (95 % CI) * | **Mean Diff.** (95 % CI) * |
| EtOH/0.9 % NaCl | EtOH/20 µM C | 8.2 (-12.7 to 29.0) | 8.3 (-9.3 to 25.8) |
| | EtOH/50 µM C | 43.1 (22.3 to 64.0) | 54.0 (36.4 to 71.5) |
| EtOH/20 µM C | EtOH/50 µM C | 35.0 (14.1 to 55.1) | 45.7 (28.2 to 63.3) |
| 0.9 % NaCl/EtOH | 0.9 % NaCl/10 µM N | 60.9 (21.2 to 100.6) * | 14.1 (-9.5 to 37.6) |
| | 0.9 % NaCl/20 µM N | 77.4 (37.7 to 117.0) * | 30.1 (6.6 to 53.7) * |
| | 20 µM C/EtOH | 0.0 (-39.7 to 39.7) | 0.0 (-23.6 to 23.6) |
| | 20 µM C/10 µM N | 42.5 (2.8 to 82.1) * | 0.7 (-22.8 to 24.3) |
| | 20 µM C/20 µM N | 63.8 (24.1 to 103.5) * | 3.8 (-22.6 to 30.1) |
| | 50 µM C/EtOH | 0.0 (-39.7 to 39.7) | 0.0 (-23.6 to 23.6) |
| | 50 µM C/10 µM N | 16.1 /-23.5 to 55.8) | -16.2 (-39.8 to 7.4) |
| | 50 µM C/20 µM N | 36.5 (-3.2 to 76.1) | -18.9 (-42.5 to 4.7) |
| 0.9 % NaCl/10 µM N | 0.9 % NaCl/20 µM N | 16.5 (-23.2 to 56.1) | 16.1 (-7.5 to 39.7) |
| | 20 µM C/EtOH | -60.9 (-100.6 to -21.2) * | -14.1 (-37.6 to 9.5) |
| | 20 µM C/10 µM N | -18.5 (-58.1 to 21.2) | -13.3 (-36.9 to 10.3) |
| | 20 µM C/20 µM N | 2.9 (-36.8 to 42.6) | -10.3 (-36.6 to 16.1) |
| | 50 µM C/EtOH | -60.9 (-100.6 to -21.2) * | -14.1 (-37.6 to 9.5) |
| | 50 µM C/10 µM N | -44.8 (-84.4 to -5.1) * | -30.2 (-53.8 to -6.7) * |
| | 50 µM C/20 µM N | -24.4 (-64.1 to 15.2) | -33.0 (-56.5 to -9.4) * |
| 0.9 % NaCl/20 µM N | 20 µM C/EtOH | -77.4 (-117.0 to -37.7) * | -30.1 (-53.7 to -6.6) * |
| | 20 µM C/10 µM N | -34.9 (-74.6 to 4.7) | -29.4 (-53.0 to -5.8) * |
| | 20 µM C/20 µM N | -13.6 (-53.2 to 26.9) | -26.4 (-52.7 to 0.0) * |
| | 50 µM C/EtOH | -77.4 (-117.0 to -37.7) * | -30.1 (-53.7 to -6.6) * |
| | 50 µM C/10 µM N | -61.2 (-100.9 to -21.6) | -46.3 (-69.9 to -22.7) * |
| | 50 µM C/20 µM N | -40.9 (-80.6 to -1.2) * | -49.1 (-72.6 to -25.5) * |
| 20 µM C/EtOH | 20 µM C/10 µM N | 42.5 (2.8 to 82.1) * | 0.7 (-22.8 to 24.3) |
| | 20 µM C/20 µM N | 63.8 (24.1 to 103.5) * | 3.8 (-22.6 to 30.1) |
| | 50 µM C/EtOH | 0.0 (-39.7 to 39.7) | 0.0 (-23.6 to 23.6) |
| | 50 µM C/10 µM N | 16.1 (-23.5 to 55.8) | -16.2 (-39.8 to 7.4) |
| | 50 µM C/20 µM N | 36.5 (-3.2 to 76.1) | -18.9 (-42.5 to 4.7) |
| 20 µM C/10 µM N | 20 µM C/20 µM N | 21.3 (-18.3 to 61.0) | 3.0 (-23.3 to 29.4) |
| | 50 µM C/EtOH | -42.5 (-82.1 to -2.8) * | -0.7 (-24.3 to 22.8) |
| | 50 µM C/10 µM N | -26.3 (-66.0 to 13.3) | -16.9 (-40.5 to 6.6) |
| | 50 µM C/20 µM N | -6.0 (-45.6 to 33.7) | -19.7 (-43.2 to 3.9) |
| 20 µM C/20 µM N | 50 µM C/EtOH | -63.8 (-103.5 to -24.1) * | -3.8 (-30.1 to 22.6) |
| | 50 µM C/10 µM N | -47.7 (-87.3 to -8.0) * | -19.9 (-46.3 to 6.4) |
| | 50 µM C/20 µM N | -27.3 (-67.0 to 12.3) | -22.8 (-49.0 to 3.7) |
| 50 µM C/EtOH | 50 µM C/10 µM N | 16.1 (-23.5 to 55.8) | -16.2 (-39.8 to 7.4) |
| | 50 µM C/20 µM N | 36.5 (-3.2 to 76.1) | -18.9 (-42.5 to 4.7) |
| 50 µM C/10 µM N | 50 µM C/20 µM N | 20.3 (-19.3 to 60.0) | -2.7 (-26.3 to 20.8) |

undiff., undifferentiated; diff., differentiated; Mean Diff., Mean Difference; CI., confidence interval; N., nimodipine; C. cisplatin
